# Non-invasive quantification of lower airway antigen-specific antibodies using exhaled breath particles – an observational proof-of-concept study

**DOI:** 10.1016/j.ebiom.2026.106538

**Published:** 2026-11-02

**Authors:** Katherina Aguilera, Andreas Hober, Nina Greilert Norin, Mina Mahdi, Vera C. Stäheli, Iryna Kolosenko, Pia Lindberg, Emma Larsson, Ali Rihani, Jessica J. Alm, Åsa M. Wheelock, Jonas Klingström, Tamás Pongrácz, Mikael Åberg, Charlotte Thålin

**Affiliations:** a Department of Clinical Sciences, Danderyd Hospital, Karolinska Institutet, Stockholm, Sweden; b Division of Immunology and Respiratory Medicine, Department of Medicine Solna, Karolinska Institutet, Stockholm, Sweden; c Department of Respiratory Medicine and Allergy, Center for Molecular Medicine, Karolinska University Hospital, Solna, Sweden; d Department of Microbiology, Tumor and Cell Biology, and National Pandemic Center, Karolinska Institutet, Solna, Sweden; e Centre for Health Crises, Karolinska Institutet, Solna, Sweden; f Department of Biomedical and Clinical Sciences, Linköping University, Linköping, Sweden; g Department of Medical Sciences, Clinical Chemistry and SciLifeLab Affinity Proteomics, Uppsala University, Uppsala, Sweden

**Keywords:** Particles in exhaled air (PExA), Lower airway immunity, Mucosal antibodies, Respiratory tract lining fluid, Immunoglobulins

## Abstract

**Background:**

Advancements in mucosal vaccine development highlight the urgent need for practical methods to assess immune responses in both upper and lower airways. Although nasal sampling is increasingly standardised, a non-invasive technique for directly assessing antigen-specific antibodies in the distal airways has been lacking.

**Methods:**

We used Particles in Exhaled Air (PExA), a non-invasive method capturing aerosolised respiratory tract lining fluid (RTLF) from the distal airways, to quantify antigen-specific IgA and IgG. Antibodies were measured in paired serum, nasal secretions, and PExA samples from 28 healthcare workers during spring 2025, including 18 with RT-qPCR-confirmed SARS-CoV-2, influenza A (H1N1), or respiratory syncytial virus (RSV) infection.

**Findings:**

Antigen-specific IgA and IgG measurements in PExA samples showed low variability (median CVs: spike-IgA 3.7%, H1-IgA 7.2%, RSV pre-F-IgA 5.3%). Strong correlations (Spearman's rho >0.85, p < 0.0001) were observed between PExA and serum IgG, which may be consistent with a systemic contribution to lower-airway IgG levels. In contrast, IgA correlations were more modest, which may reflect local IgA production in the lungs. IgG and IgA correlated between PExA and nasal secretions, while the IgA/IgG ratio was higher in nasal secretions (median 5.0 vs 0.6, p < 0.0001), consistent with expected isotype distribution in upper and lower airways.

**Interpretation:**

This study demonstrates the feasibility of non-invasive measurement of antigen-specific antibodies in the lower airways. Our approach proposes a framework for assessing lower-airway immunity and supporting next-generation mucosal vaccine development.

**Funding:**

This study was funded by the European Research Council, the Swedish Society for Medical Research, and the Knut and Alice Wallenberg Foundation.

**Research in context**

**Evidence before this study**

The rapidly expanding mucosal vaccine pipeline underscores an urgent need for practical, non-invasive approaches to assess immune responses across both the upper and lower airways. While nasal lining fluid is easily accessible, sampling from the lower airways typically requires invasive bronchoscopy. We searched PubMed from database inception to March 2025 using the following combination of search terms: (antibody OR antibodies) AND (“mucosal immune” OR “mucosal immunity”) AND (“lower airway” OR “distal airways” OR lung OR respiratory) with no other inclusion/exclusion criteria, supplemented by screening the reference lists from relevant publications.

Existing studies exploring antigen-specific antibodies in the lower airways have relied on bronchoscopy or bronchoalveolar lavage, and evidence on non-invasive assessment of antigen-specific immunity in the distal airways remains limited.

**Added value of this study**

This study demonstrates that antigen-specific antibodies can be quantified non-invasively in distal airway lining fluid using particles in exhaled air (PExA). Combining this sampling method with highly sensitive electrochemiluminescence immunoassays, we demonstrate precise and reproducible measurement of virus-specific IgA and IgG in the lower airways following SARS-CoV-2, influenza A virus, and respiratory syncytial virus infections. Antigen-specific IgG levels in PExA samples strongly correlated with serum levels, whereas IgA correlations were more modest, which may reflect a transudation of IgG from blood to the lungs and a localised mucosal IgA generation. An IgG dominance in PExA samples, as opposed to an IgA dominance in nasal secretions, furthermore, reflects the expected immunological gradient across upper and lower airways.

**Implications of all the available evidence**

Non-invasive assessment of antigen-specific immune responses in the lower airways has the potential to address a major methodological gap in respiratory immunology. If confirmed in larger studies, PExA-based sampling could enable broader investigation of lung-specific immunity and provide a valuable research tool for studying compartmentalised respiratory immunity and antibody kinetics in the lungs. Further work is needed to establish its broader applicability, functional relevance, and potential translational value.

## Introduction

Antibodies in the respiratory mucosa are a fundamental component in the protection against airborne pathogens. While currently employed parenteral vaccines against respiratory viruses protect against severe disease, they provide limited protection against infection and onward transmission^1^, ^2^ as they fail to elicit robust antibody responses at the site of viral entry.^3^, ^4^ This limitation has prompted intense global efforts to develop mucosal vaccines that induce both systemic and local immunity through intranasal or inhaled antigen delivery^5^, ^6^, ^7^ (reference 6 is not peer-reviewed). However, evaluations of such vaccines are constrained by the lack of practical methods for assessing mucosal immune responses, particularly in the lower airways. While sampling from the upper airways through non-invasive nasal swabs or nasosorption devices is becoming increasingly standardised,^8^, ^9^, ^10^ accessing the lower respiratory tract poses specific challenges as it requires invasive bronchoscopy and bronchoalveolar lavage (BAL).^11^, ^12^ Non-invasive approaches, such as induced sputum, primarily reflect secretions from the larger, more proximal airways and may be influenced by contamination from the upper airways and do not specifically capture the distal airway compartment. Very little is therefore known about antibody responses in the lower airways, despite their relevance at the site of pathology in severe respiratory infections.

Building on our validated protocols for antibody quantification in nasal secretions,^4^, ^8^, ^9^, ^13^, ^14^, ^15^, ^16^, ^17^ we evaluated Particles in Exhaled Air (PExA) for non-invasive measurement of antigen-specific antibodies in respiratory tract lining fluid (RTLF). As shown in previous studies^18^, ^19^, ^20^ and summarised in Supplementary Table S1, PExA samples are generated during the reopening of collapsed small airways and originate predominantly from the distal airways. Our primary objective was to assess the feasibility and precision of quantifying antigen-specific IgA and IgG in PExA samples. We further compared antibody levels in PExA with paired serum and nasal secretions to explore their distribution across respiratory and systemic compartments.

## Methods

### Study population

Participants were enrolled through the longitudinal COMMUNITY healthcare worker cohort (NCT06784739), which investigates immune responses to respiratory infections and vaccinations. In April 2020, a total of 2149 healthcare workers were recruited at Danderyd Hospital in Stockholm, Sweden. Since enrolment, blood, saliva, and nasal secretion samples have been collected every four months, alongside vaccination and infection data obtained from Swedish national registers and reverse-transcription quantitative polymerase chain reaction (RT-qPCR) screening programs. Participants were provided with at-home sampling kits for self-administered nasal, oropharyngeal, and saliva swabs and were instructed to self-sample upon symptoms of respiratory infection or on a weekly basis during periods of high viral spread. The samples were analysed at the National Pandemic Centre (NPC) at Karolinska Institutet using RT-qPCR within 48 h of collection, as detailed in the Supplementary Material.

Participants were selected based on a home-based PCR screening program during the spring of 2025. A total of 18 participants with RT-qPCR-confirmed SARS-CoV-2, influenza virus, and/or RSV infection were included in the study. Eight participants had influenza A (H1N1) infection, two had influenza B infection, four had RSV infection, and two had SARS-CoV-2 infection. Two additional participants experienced sequential infections, one with SARS-CoV-2 and influenza A virus (three months apart) and one with RSV and SARS-CoV-2 (one month apart). Participants with sequential infections contributed to each infection-specific analysis using samples collected before and after the corresponding infection. In addition to infected participants, 10 participants without known infection in the previous six months were included from the COMMUNITY cohort, rendering a total of 28 study participants. The study design is presented in Fig. 1. For analyses exploring infection-induced antibodies (Fig. 2), participants infected with influenza B (n = 2) and participants with a positive PCR test at baseline (n = 1; positive SARS-CoV-2) were excluded. All 28 participants contributed to analyses of precision of antigen-specific antibody measurements in PExA samples (Fig. 3), correlations between antigen-specific antibody levels in PExA samples, nasal secretions, and serum (Fig. 4), and total IgA-to-IgG ratio in nasal secretions and in PExA samples (Fig. 5). Baseline serum and nasal secretion samples were obtained from the regular follow-up visit prior to infection in December 2024. Serum and nasal secretions were sampled at 30 and 90 days after RT-qPCR-confirmed infection. PExA samples were collected in May 2025, which coincided with the one-month follow-up after SARS-CoV-2 infections and the three-month follow-up after influenza A and RSV infections (Fig. 1A). Some participants did not provide samples at one or more follow-up time points, resulting in varying numbers of available samples at day 30 and day 90. Analyses were based on available samples at each time point. Participant demographics are summarised in Supplementary Table S2. Information on biological sex was obtained from participants’ electronic medical records and was recorded as female or male.

**Fig. 1:**
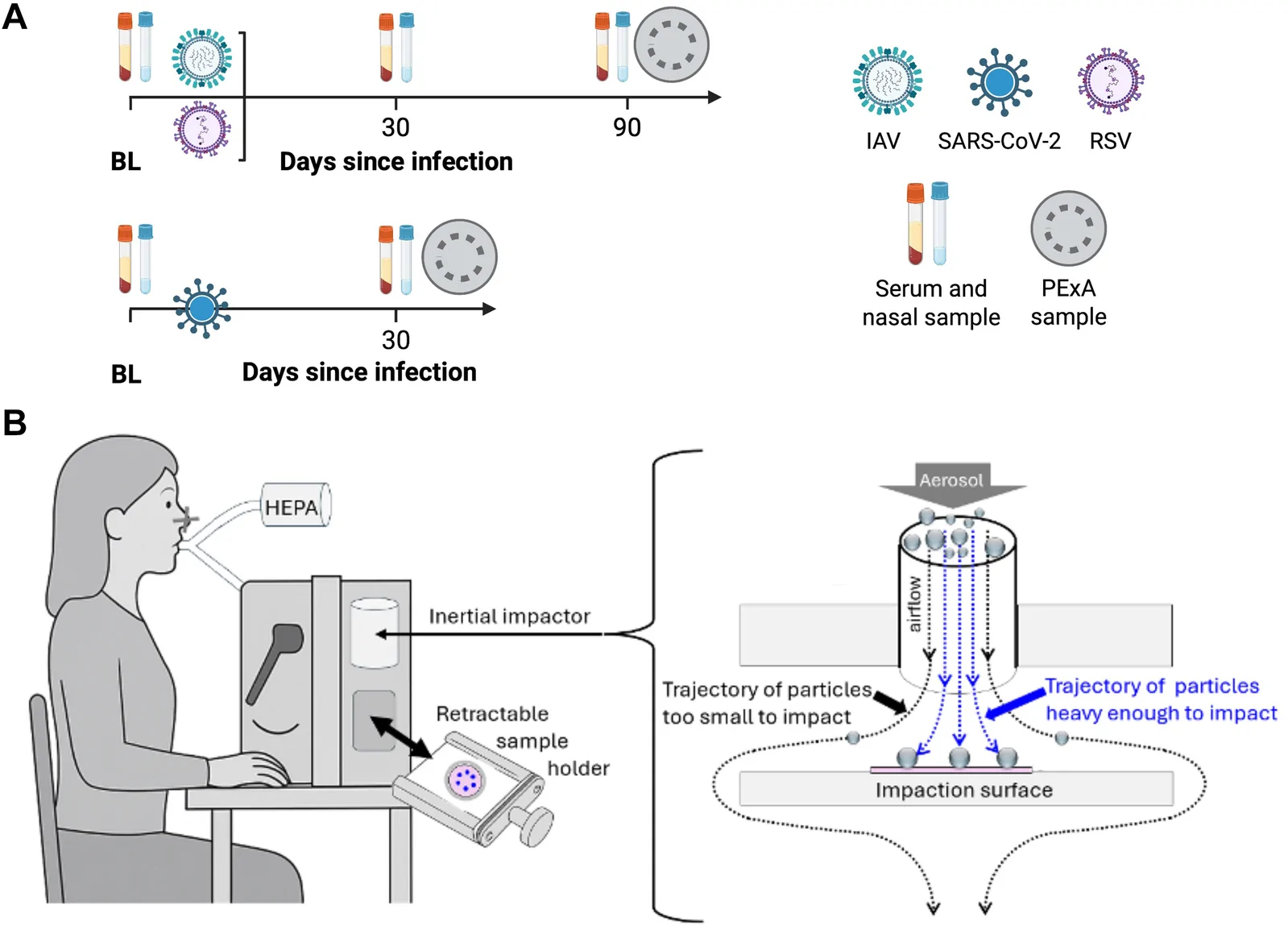
**Study design and collection of lower respiratory tract lining fluid (RTLF) using the PExA instrument.** A) Study design and sample collection: Serum, nasal secretions, and PExA samples were collected from participants enrolled in the ongoing COMMUNITY cohort study. Serum and nasal secretions were collected prior to, and at 30 and 90 days post-infection. PExA samples were collected at the post-infection sampling time point. Serum, nasal secretions, and PExA samples were also collected from 10 participants without known infection in the previous 6 months. A detailed flow chart of study participants is provided in Supplementary Fig. S1. B) Participants inhaled high efficiency particulate arresting (HEPA) filtered air and performed a standardised breathing manoeuvre consisting of airway closure, reopening, and exhalation into the PExA instrument. As distal airways reopen from a closed state, a phospholipid lining fluid film forms between the opposing luminal airway surfaces. This film ruptures as the airways continue to expand, generating an aerosol of droplets originating from the lower airway RTLF. According to Newton's first law, particles with sufficient inertia cannot follow the deflected airflow and therefore impact the collection surface, while smaller particles follow the streamlines. The exhaled particles were deposited onto a substrate mounted in a retractable sample holder for subsequent molecular analysis. RTLF, respiratory tract lining fluid; BL, baseline; IAV, Influenza A virus; RSV, respiratory syncytial virus; PExA, Particles in Exhaled Air.

**Fig. 2:**
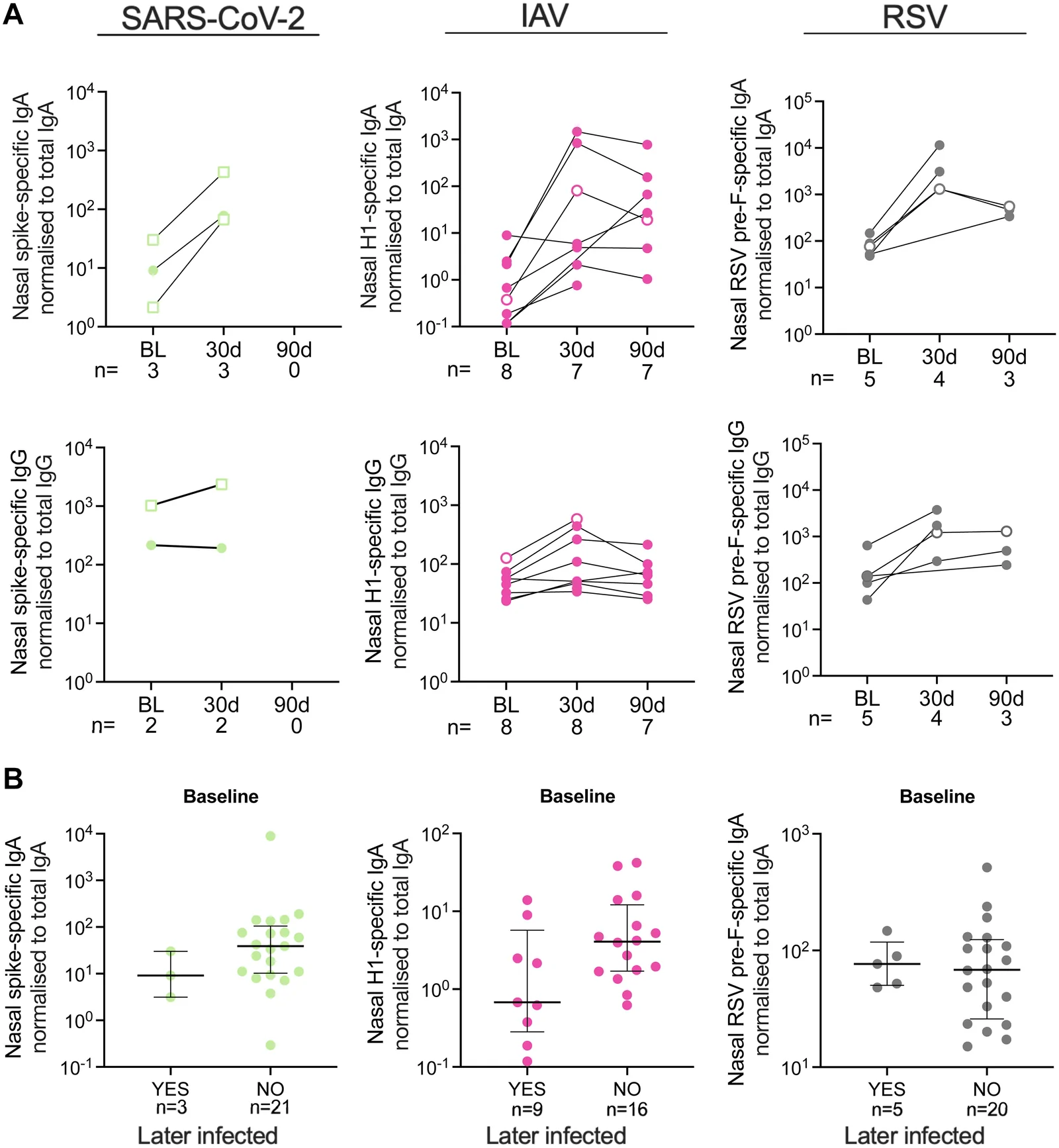
**Antigen-specific antibody responsesfollowing SARS-CoV-2, influenza A virus or RSV infection and prior to RT-qPCR screening.** (A) Levels of antigen-specific IgA and IgG were measured over time in nasal secretions collected before infection (baseline, BL), and at 30 and 90 days post-infection. Sample sizes vary across time points because not all participants provided samples at all time points. (B) BL nasal IgA levels measured in participants who later became infected with SARS-CoV-2, influenza A virus, or RSV and in participants who did not become infected with the respective virus. Nasal antigen-specific IgA and IgG (AU/mL) were normalised to total IgA and IgG (ng/mL) in the same sample, multiplied by 10^7^ and presented as a ratio. Horisontal solid lines represent medians, and error bars represent the IQR. No further statistical analysis was performed on these observations due to the limited sample size. Open circles indicate the first infection in participants with sequential infections, and open squares indicate the second infection. Data represent medians of two aliquots from the same sample. SARS-CoV-2, severe acute respiratory syndrome coronavirus 2; IAV, Influenza A virus; RSV, respiratory syncytial virus.

**Fig. 3:**
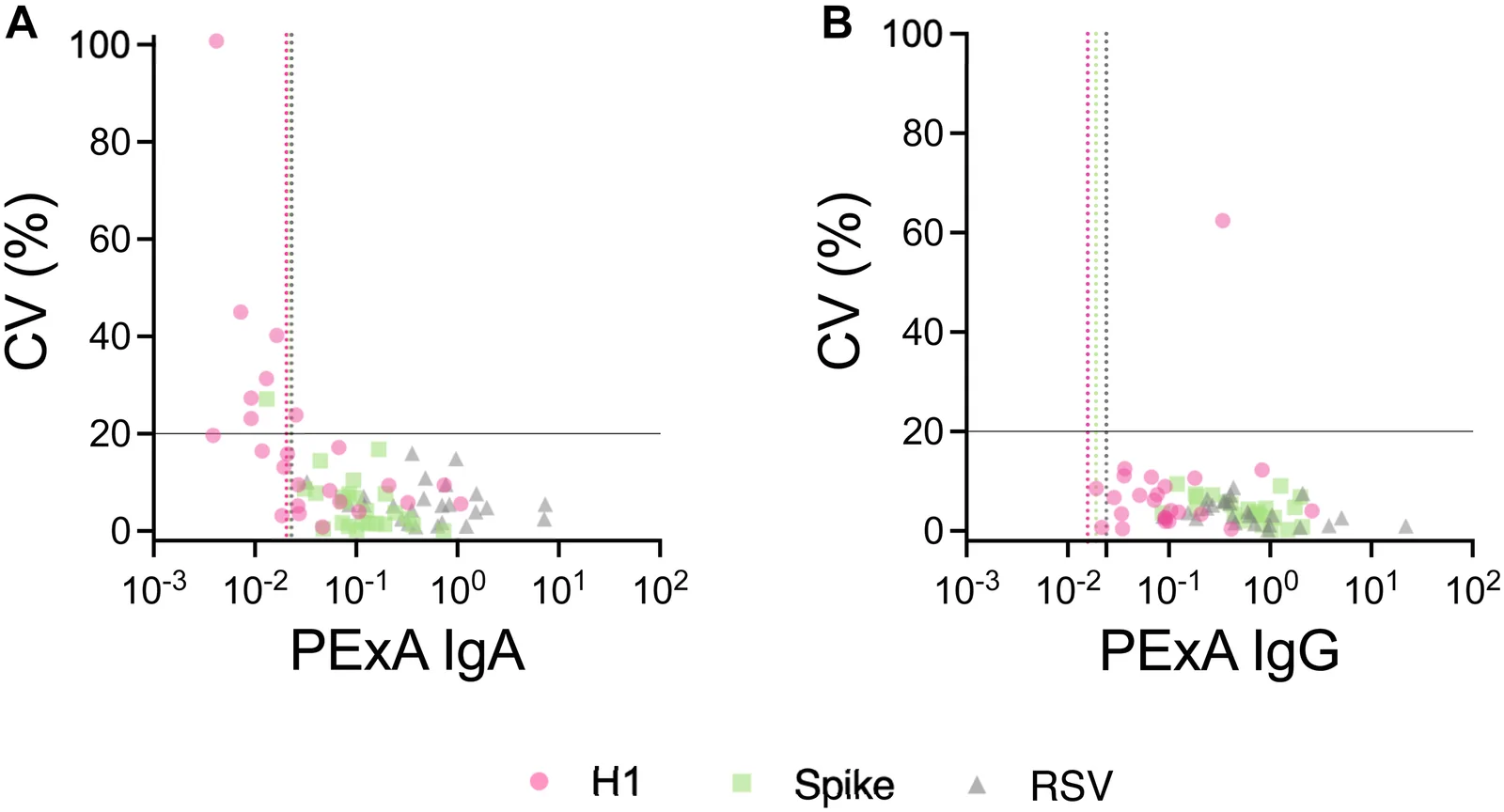
**Precision of antigen-specific antibody measurements in PExA samples.** CVs for antigen-specific IgA (A) and IgG (B), calculated from technical duplicates (n = 24) using the same extracted non-normalised sample. Summary statistics (median and IQR) were calculated using only samples with antigen-specific antibody levels above the limit of detection (LOD); samples below the LOD were excluded from these calculations. H1-specific antibodies are shown in pink, spike-specific antibodies are shown in green, and RSV pre-F-specific antibodies are shown in grey. Antigen-specific LODs are indicated by dashed lines: pink for H1, green for spike, and grey for RSV pre-F. Horisontal lines represent 20% CV. CV, coefficient of variation.

**Fig. 4:**
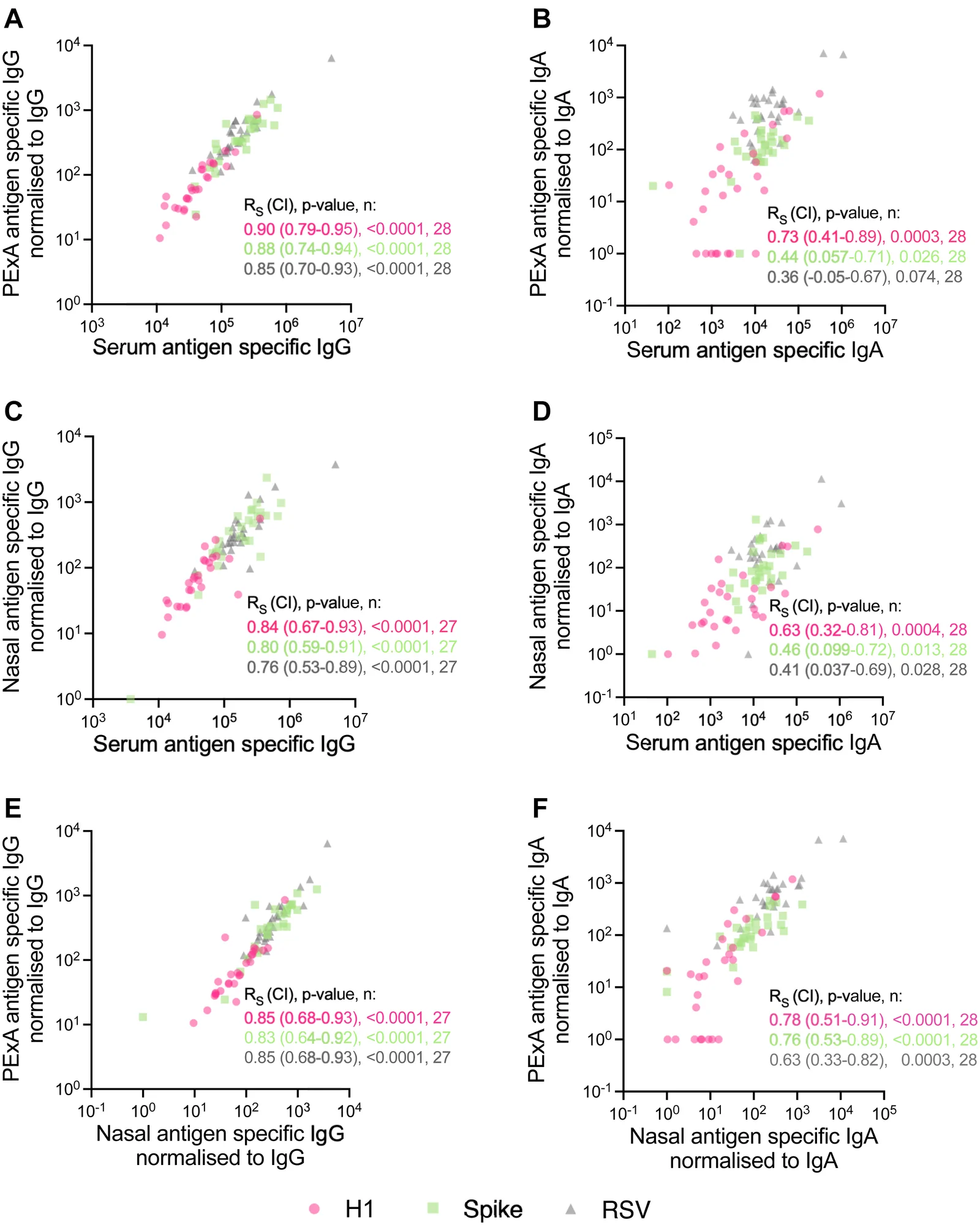
**Correlations between antigen-specific antibody levels in PExA samples, nasal secretions and serum.** A) Correlations between antigen-specific IgG in PExA samples and in serum. B) Correlations between antigen-specific IgA in PExA samples and in serum. C) Correlations between antigen-specific IgG in nasal secretions and in serum. D) Correlations between antigen-specific IgA in nasal secretions and in serum. E) Correlations between antigen-specific IgG in matched PExA samples and nasal secretions. F) Correlations between antigen-specific IgA in matched PExA samples and nasal secretions. Nasal and PExA antigen-specific IgA and IgG (AU/mL) were normalised to total IgA and IgG (ng/mL) in the same sample, multiplied by 10^7^, and presented as a ratio. Data represents medians of two aliquots from the same sample. Correlations were assessed by Spearman's rank correlation. Spearman's correlation coefficients (R_S_), 95% confidence intervals (CI), and corresponding p-values are shown in the figure.

**Fig. 5:**
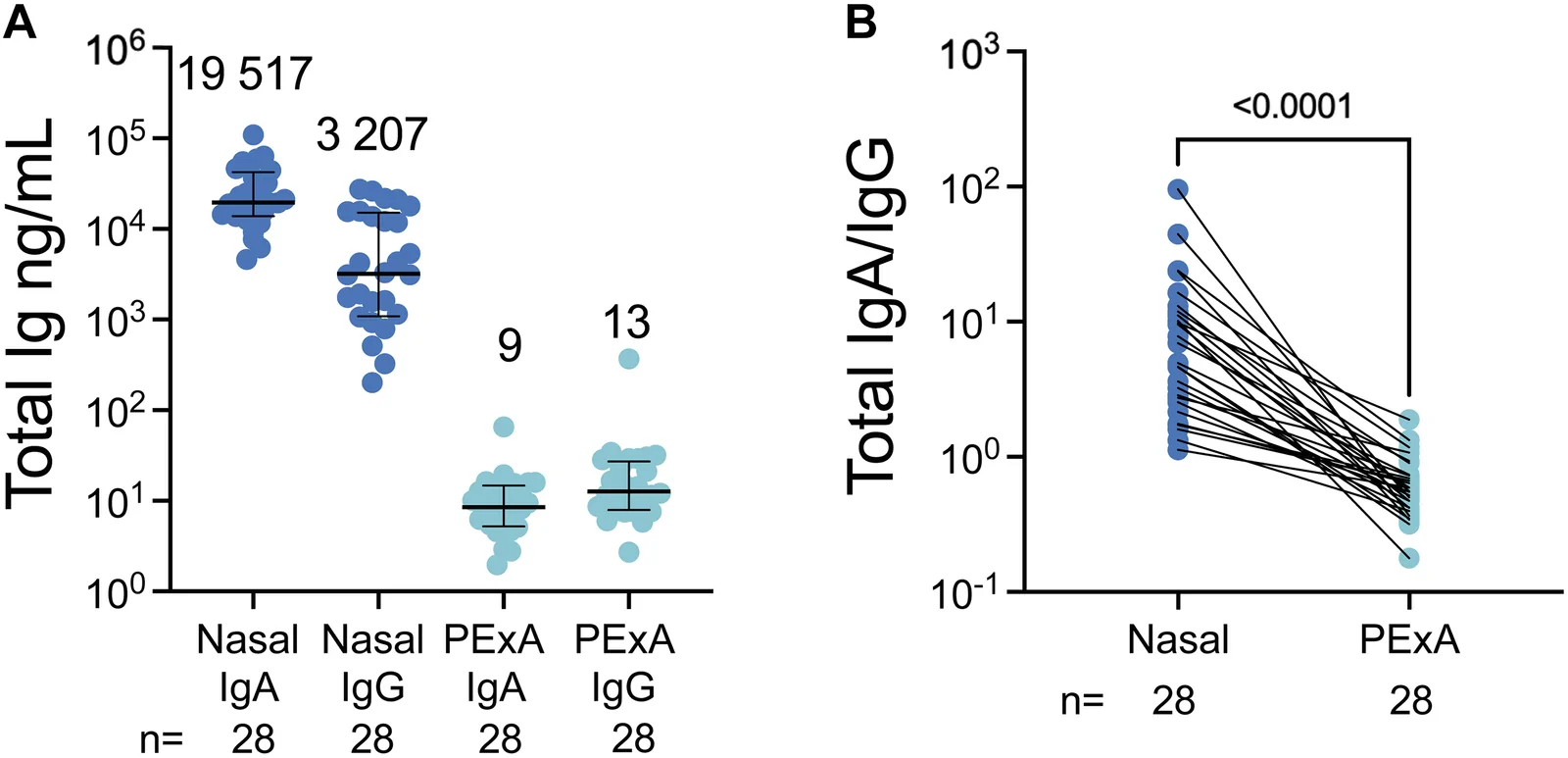
**Total Ig and IgA-to-IgG ratio in nasal secretions and PExA samples.** A) Total IgA and IgG in paired nasal secretions and PExA samples. Points represent individual participants. Horisontal solid lines represent the median, and error bars represent the IQR. Values represent medians. B) Total IgA-to-IgG ratio in nasal secretions and in PExA samples. Data represents medians of two aliquots from the same sample. Pairwise comparison of the IgA-to-IgG ratio was conducted using a Wilcoxon signed-rank test.

### Ethics

All participants provided written informed consent, and the study protocol was approved by the Swedish Ethical Review Authority (dnr 2020-01653 and 2025-03248-02). The study was conducted in accordance with the ethical principles outlined in the Declaration of Helsinki (2024 revision).

### Nasal secretion sampling

The soft tip of a FLOQSwab (catalogue no. CP519CS01, COPAN Diagnostics Inc., USA) was inserted into the anterior nostril and rotated five times to ensure contact with the nasal mucosa. The swab was then transferred to a 15 mL centrifuge tube containing 1 mL of 1× Dulbecco's phosphate-buffered saline (PBS; catalogue no. 14190-250, Gibco). The tubes were vortexed for 10s, after which the swab was removed. The resulting solution was then aliquoted and stored at −80 °C.

### Serum sampling

Blood samples were obtained by venipuncture into serum separator tubes (7/5 mL SST II, BD Vacutainer®). Samples were allowed to clot for at least 30 min at room temperature before being centrifuged at 2000×*g* for 10 min. The resulting serum was then aliquoted and stored at −80 °C within 1 h for subsequent analyses.

### PExA sampling

Lower RTLF was collected using the PExA instrument (v. 2.1, PExA AB, Gothenburg, Sweden), according to the manufacturer's instructions (Fig. 1B) and as previously described.^21^ Briefly, study participants wore a nose clip and breathed into the PExA instrument via a mouthpiece and a two-way, non-rebreathing valve, inhaling HEPA-filtered air to remove particles from ambient air. Exhaled particles with diameters between 0.4 and 7 μm were counted by an optical particle counter (Grimm Aerosol Technik GmbH & Co, Ainring, Germany) in the instrument. The particle mass was estimated by the instrument software based on the measured particle number and size distribution. Particles were collected by impaction on dried blood spot (DBS) membranes (Whatman 903 Protein saver, specially tailored for PExA, PExA AB). A standardised breathing manoeuvre was used, starting with an exhalation at normal flow rate to residual volume, followed by breath holding for 5s, then a maximal inhalation to total lung capacity, and finally a normal exhalation to functional residual capacity. Particle collection was activated at the point of full inhalation, and the breathing manoeuvre was repeated until 240 ng of exhaled particles had been collected or until the subject was no longer willing or able to continue with the sampling. After collection, ten punches were excised from the collection substrate and extracted in PBS containing 0.1% Tween 20 (P1379, Sigma–Aldrich). To account for variation in collected particle mass between samples, the volumes were adjusted to a final concentration of 4.8 ng particles per μL. The mixture was incubated at 37 °C with shaking (700 rpm) for 1 h. Following extraction, the substrate punches were transferred into mesh-supported tube inserts, which were placed into collection tubes and sealed. The tubes were centrifuged at 10,000×*g* for 1 min, and the supernatant was collected and stored at −80 °C until further analysis.

The PExA technology and instrumentation (PExA v. 2.1) are patented and commercially available from PExA AB (Gothenburg, Sweden). All consumables, sampling parameters, and operating procedures required to replicate the sampling procedure are provided with the instrument or described herein. No additional proprietary restrictions apply beyond the existing patent protection covering the technology itself.

### Quantification of antigen-specific IgA and IgG

Antigen-specific IgA and IgG were quantified in nasal secretions (optimised dilution 1:1000), serum (optimised dilution 1:50000), and PExA samples (optimised dilution 1:4) using the V-PLEX Respiratory Prototype Panel 4 (Meso Scale Diagnostics; MSD, Maryland, USA) according to the manufacturer's instructions. The specific antigen targets included wild-type SARS-CoV-2 spike protein, influenza A (H1N1, strain A/Wisconsin/67/2022) haemagglutinin (HA) protein, and RSV pre-fusion (pre-F) protein. Influenza A H1N1-specific antibodies were measured using a prototype COVID-Flu-RSV panel developed and qualified by MSD, while SARS-CoV-2 wild-type (WT) antibodies were analysed using the MSD SARS-CoV-2 Panel 31 (catalogue no. K15642U for IgG and K15644U for IgA), according to the manufacturer's instructions. Reference Standard 1 (catalogue no. C00ADK-2), a serum-based calibrator quality-controlled by MSD, was used for calibration. Seven-point standard curves were prepared using four-fold serial dilutions and fitted using a 4-parameter logistic model. Each calibrator level was analysed in duplicate together with a blank (zero calibrator), with acceptance criteria of ≤20% CV between duplicates and 80–120% recovery of nominal concentrations. Samples were analysed in duplicate. Detection was performed using SULFO-TAG-conjugated mouse monoclonal anti-human IgG (catalogue no. D21ADF) or anti-human IgA (catalogue no. D21ADE) antibodies supplied with the kits. Antibody concentrations were interpolated from the corresponding standard curves, and final concentrations in undiluted samples were calculated after correction for sample dilution.

Total IgA and IgG concentrations were determined in nasal secretions and PExA samples using the Isotyping Panel 1 Human/NHP Kit (MSD, catalogue no. K15203D), following the manufacturer's protocol. Optimised dilutions used were 1:5000 for total IgA in nasal secretions and 1:10 in PExA, and 1:500 for total IgG in nasal secretions and 1:10 in PExA. All plates were analysed using an electrochemiluminescence reader (MESO SECTOR S600 instrument, MSD). To account for potential variability in sample collection and dilutions, antigen-specific IgA and IgG levels were normalised to total IgA and total IgG levels within the same sample. The resulting ratios were multiplied by 10^7^ for visualisation purposes. The limit of detection (LOD) for nasal and PExA samples was set using the Discovery Workbench 4.0 Analysis Software (Meso Scale Diagnostics; MSD, Maryland, USA) and determined as the mean signal of the blank (the lowest calibrator curve point) plus 2.5 SD according to the manufacturer's instructions.

### Statistical analyses

No formal sample size calculation was performed, as this was an exploratory observational study and all eligible participants in the COMMUNITY cohort (i.e. participants with RT-qPCR-confirmed SARS-CoV-2, influenza, or RSV infection during the study period) were invited to participate. Ten participants without known infection during the previous six months were randomly selected from the COMMUNITY cohort for comparison. Participants with infections other than SARS-CoV-2, influenza, or RSV, as well as participants with a positive PCR test at baseline, were excluded from the infection-induced antibody analyses. Randomisation and blinding were not applicable. Analyses were performed using available data only, and missing samples were excluded from the relevant analyses without imputation. No adjustment for potential confounders or multiple comparisons was performed, as all analyses were exploratory. Participants with sequential infections contributed to each infection-specific analysis using samples collected following the corresponding infection. Nasal (n = 1) and PExA (n = 8) samples with antigen-specific IgA or IgG levels below the LOD, but with measurable concentrations of the corresponding total immunoglobulin, were assigned a value of 1 to allow their inclusion in the analyses. This value was below the lowest detectable concentrations of nasal and PExA antigen-specific IgG and IgA. Nasal samples with total IgG or IgA levels below the LOD (n = 1) were excluded from the relevant analyses. Total IgG and IgA were above LOD in all PExA samples. No measurements exceeded the upper limit of quantification (ULOQ). Coefficients of variation (CVs) were defined as the ratio of the standard deviation to the mean and calculated from technical duplicates using the same extracted non-normalised sample. Correlations were assessed by Spearman's rank correlation. Wilcoxon signed-rank tests were used for the paired comparisons of the IgA-to-IgG ratio in nasal secretions and PExA samples. As a robustness check, a sign test (two-sided binomial test with p = 0.5) was also performed. Exact p-values are reported with two significant digits, and values smaller than 0.0001 are reported as <0.0001. All statistical analyses were performed using GraphPad Prism (v. 10.0.3).

### Role of the funders

The funding sources were not involved in the study design, data collection, data analysis, interpretation of the findings, or preparation of the manuscript. The authors retained full independence throughout the research process and were solely responsible for the decision to submit the manuscript for publication.

## Results

We measured antigen-specific IgA and IgG in paired blood, nasal secretions, and PExA samples from 18 healthcare workers (HCWs) following SARS-CoV-2, influenza A/B, and/or RSV infection, as well as from 10 HCWs without known infection in the preceding 6 months.

Among the 28 participants, 26 (93%) were of the female sex, with a mean (SD) age of 50 (11) years. All participants had been previously vaccinated against SARS-CoV-2, with a mean (SD) of 1320 (84) days since vaccination. Fifteen participants (54%) had also received influenza vaccination, with a mean (SD) of 305 (148) days since vaccination. Participant characteristics stratified by infection status are summarised in Supplementary Table S2.

Blood samples and nasal secretions were available both before and after infection. Nasal antigen-specific IgA levels increased after infection (Fig. 2A). Median levels increased 9-fold 30 days post SARS-CoV-2 infection (from 9.1 (interquartile range (IQR) 3.1–30.2) to 77.9 (IQR 66.7–428.5)), 12-fold 30 days post influenza A virus infection (from 0.5 (IQR 0.1–2.4) to 5.9 (IQR 2.1–839.1)) and 29-fold 30 days post RSV infection (from 76.7 (IQR 50.2–118.3) to 2204 (IQR 1312–9421)). Moderate increases were also observed in nasal antigen-specific IgG levels (675.9 (IQR 327.2–1025) to 1419 (IQR 477.9–2361)). Baseline nasal antigen-specific IgA levels were lower in participants who later became infected with SARS-CoV-2 or influenza A virus (9.1 (IQR 3.1–30.2), 0.68 (IQR 0.28–5.72)) compared with those who did not become infected with the respective virus (39.0 (IQR 10.2–105.2), 4.08 (IQR 1.70–12.14)) (Fig. 2B).

The PExA instrument was available only at the post-infection time point, and paired blood, nasal, and PExA samples were collected 1–3 months post-infection. There was a low degree of variation between antigen-specific antibody measurements in duplicate PExA samples with titres above the LOD (Fig. 3). The median and IQR CV was 3.7% (1.4%–7.7%) for spike-specific IgA, 7.2% (4.9%–11.1%) for H1-specific IgA, and 5.3% (2.5%–7.6%) for RSV pre-F-specific IgA. Even lower CVs were observed for antigen-specific IgG, with median and interquartile range (IQR) CVs of 4.2% (2.8%–6.6%) for spike-specific IgG, 5.2% (2.6%–10.2%) for H1-specific IgG, and 3.2% (1.5%–6.0%) for RSV pre-F-specific IgG, demonstrating high assay precision across isotypes and antigen specificities.

There were strong correlations between antigen-specific IgG in matched PExA and serum samples (Fig. 4A). In contrast, correlations were more modest between antigen-specific IgA levels in matched PExA and serum samples, with modest but significant correlations observed for SARS-CoV-2 and influenza A, whereas the RSV-specific correlation did not reach statistical significance (Fig. 4B). Similar patterns were observed when comparing antigen-specific antibodies in matched nasal secretions and serum samples, with consistently stronger correlations for IgG than for IgA (Fig. 4C and D).

We next compared antigen-specific antibody levels in the upper and lower airways using matched nasal secretions and PExA samples. Strong correlations were found between spike-specific, H1-specific, and RSV pre-F-specific IgA and IgG responses in matched nasal secretions and PExA samples. The correlations were consistently stronger for IgG as compared to IgA (Fig. 4E and F).

The concentrations of total IgA and IgG were substantially higher in nasal secretions as compared with PExA samples, with median (IQR) of 19,517 (3902–42,290) and 8.54 (5.23–14.80) ng/mL IgA, respectively, and 3207 (1087–15,092) and 12.73 (7.94–27.11) ng/mL IgG, respectively (Fig. 5A). Notably, the total IgA-to-IgG ratios confirmed the mucosal gradient, with a greater IgA dominance in nasal secretions (median ratio of 4.97 (IQR 2.55–11.93)) as compared to PExA samples (median ratio of 0.56 (IQR 0.42–0.74)) (Fig. 5B).

As a robustness analysis, the paired sign test showed that all 27 paired differences were positive (n = 27 paired comparisons; p < 0.0001), consistent with the primary analysis. The median paired difference was 4.30 (95% CI 2.13–10.02).

## Discussion

This study demonstrates that antigen-specific antibodies can be quantified non-invasively in RTLF from the distal airways using PExA. By overcoming key barriers to lower-airway sampling, this approach establishes a practical means of studying mucosal immunity and evaluating vaccines designed to induce mucosal immune responses in both upper and lower airways.

The need to develop innovative vaccines capable of inducing not only systemic but also strong mucosal immune responses in both the upper and lower respiratory tracts is evident, as shown by the rapid innovation in mucosal vaccine development driven by the COVID-19 pandemic.^5^, ^7^ As of July 2025, at least 28 mucosal COVID-19 vaccines are in clinical evaluation,^5^ and five vaccines have received approval for human use in China, Russia, India, Iran, and Indonesia.^7^ Building on the foundation of the three licensed intranasally delivered live-attenuated influenza vaccines, at least 13 new mucosal influenza vaccines are currently in clinical trials^6^ (not peer-reviewed). Robust evidence on their ability to induce mucosal immune responses in humans, however, remains scarce and has primarily focused on analyses of saliva or nasal secretions, which may not reflect antibodies in the lower respiratory tract.

The respiratory tract comprises upper and lower regions, each characterised by unique immune features,^22^ which limits extrapolation of immune responses from the upper to the lower airways. Importantly, upper and lower airway responses also depend on the route of antigen delivery.^23^ Intramuscular vaccination primarily elicits IgG responses in the lower airways through transudation from the circulation. In contrast, mucosal infections or vaccines also induce secretory IgA in the respiratory mucosa by activating local tissue-resident plasma cells.^24^, ^25^ Moreover, intranasal vaccines typically generate droplets measuring 30–100 μm, which primarily deposit in the upper airways, whereas aerosolised delivery produces droplets smaller than 5 μm that can penetrate to the lower respiratory tract and lungs.^26^ Reflecting this, a recent study comparing mucosal immunogenicity of an intranasal or aerosolised vaccine in non-human primates demonstrated that aerosol delivery enhanced anti-spike IgA responses in the lower airways. In contrast, intranasal administration preferentially boosted IgA in the upper airways.^23^ Moreover, neutralisation in nasal washes was primarily IgA-dependent following intranasal vaccination, while IgG predominated in the lower airways, emphasising that correlates of protection against infection likely differ between the upper and lower airways. Collectively, this underscores that preventing infection and controlling disease rely on distinct antibody responses, and that a comprehensive evaluation of both upper and lower airway immune responses is essential for defining optimal mucosal vaccine strategies.

As supported by earlier observations following SARS-CoV-2 and influenza infections,^16^, ^27^, ^28^ we observed an increase in antigen-specific IgA levels in nasal secretions following infections with SARS-CoV-2, influenza A virus, and RSV. These findings are consistent with previous work showing early mucosal IgA responses following SARS-CoV-2 infection,^13^ influenza A H3N2 virus infection,^29^ human influenza A H1N1 virus challenge studies,^30^ and RSV infection.^31^ Given the limited subgroup sample sizes, particularly for SARS-CoV-2, these fold-change estimates should be considered descriptive observations within this cohort. Although we were only able to obtain PExA samples from the last follow-up and were therefore not able to assess infection-induced antibody responses in these samples, the elevation of antigen-specific IgA in nasal secretions together with the strong correlations between antigen-specific antibodies in PExA samples and nasal secretions may be consistent with similar antibody responses in the lower airway following respiratory infection. Interestingly, baseline levels of antigen-specific IgA in nasal secretions were lower in participants who subsequently became infected with either SARS-CoV-2 or influenza A virus compared with participants who remained uninfected during follow-up. However, no statistical analysis was performed on these observations due to the limited sample size. Accordingly, the findings should be considered an exploratory proof-of-concept demonstrating the feasibility of detecting antigen-specific antibodies in lower airway samples using PExA, rather than a correlate of protection analysis. Additionally, it is worth noting that the participants in this study were from a healthy cohort of Swedish healthcare workers, and it would be of interest to conduct follow-up studies in a broader population. However, the well-defined nature of the cohort allowed us to thoroughly verify the infection history for the participants. Importantly, the observed trend is in line with earlier observations that mucosal antibody levels serve as markers of protection against respiratory infection.^16^, ^27^

The low CVs for both antigen-specific IgG and IgA in PExA samples support the robustness and high precision of antigen-specific antibody measurements using this non-invasive method. Notably, IgG measurements showed slightly lower CVs than IgA, likely due to higher concentrations of IgG in the lower airways.^25^ Importantly, a limitation of the PExA method is the small volume of RTLF that is collected. The device captures distal airway particles ranging from 0.4 to 7 μm,^21^ which are generated during airway closure and reopening. While previous studies suggest that the collected material reflects fluid from the small airways, the total volume obtained is minute, necessitating highly sensitive and well-validated assays for reliable quantification of antigen-specific antibodies. This is supported by our data, which show that sensitive assays, such as the employed electrochemiluminescence-based assay, can reliably quantify antigen-specific antibodies in PExA samples.

Interestingly, we observed strong correlations between antigen-specific IgG responses in matched blood and PExA samples across all tested antigen specificities, whereas correlations between antigen-specific IgA in matched blood and PExA samples were more modest. Although these correlations do not establish the origin of the detected antibodies, this may reflect an IgG transfer from serum through the lung epithelial lining even in the absence of inflammation^24^, ^25^, ^32^, ^33^ and a compartmentalised nature of IgA production locally at mucosal surfaces by tissue-resident plasma cells.^34^ Furthermore, because many participants were vaccinated against SARS-CoV-2 and influenza, the observed IgG responses likely reflect a combination of vaccine-induced and infection-induced immunity, and the relative contribution of these exposures cannot be determined in the present study.

As expected, following respiratory infection,^35^ antigen-specific IgA and IgG levels correlated between matched nasal and PExA samples. Correlations were stronger for IgG, which may reflect its systemic origin and uniform distribution along the respiratory tract, whereas IgA correlations were less pronounced and may be consistent with local mucosal IgA responses, highlighting the importance of assessing both upper and lower airways. Although correlations between nasal secretions and PExA samples could suggest potential upper-airway contamination, extensive biochemical and proteomics analyses have provided strong evidence that PExA samples originate from the small airways.^18^, ^20^, ^36^, ^37^, ^38^, ^39^, ^40^, ^41^ An extensive summary of the findings from studies validating the origin of the PExA samples is provided in Supplementary Table S1. Proteomics analyses using liquid chromatography–mass spectrometry (LC-MS) on pooled PExA material from several individuals have revealed protein profiles with up to 80% similarity to bronchoalveolar lavage fluid (BALF).^18^ Moreover, surfactant protein A (SP-A), a highly abundant pulmonary protein, can be reliably quantified in PExA samples, exhibiting low intra-individual variability and a strong correlation with SP-A levels in BALF.^40^, ^42^ Previous studies have also demonstrated that amylase, the most abundant protein in saliva, is absent among the proteins identified in PExA samples^18^, ^43^; (reference 42 is not peer-reviewed landscape review). The greater IgA dominance in nasal secretions compared to in PExA samples in our study further supports a lower airway origin of the PExA samples, aligning with prior knowledge that IgA is the dominant immunoglobulin at mucosal surfaces of the upper respiratory tract, whereas IgG dominates the lower airways.^25^, ^33^, ^44^, ^45^ It should be noted, however, that the lower airway origin of the PExA samples was not directly verified in the present study. The present study focused exclusively on viral respiratory infections (SARS-CoV-2, influenza A virus, and RSV), and immune responses in the lower airways may differ between viral and bacterial infections due to differences in inflammatory responses, epithelial permeability, and immunoglobulin dynamics. Therefore, the applicability of this approach to bacterial respiratory infections remains to be established and should be addressed in future studies.

A limitation of the present study is that the analyses were restricted to antibody levels and did not include an assessment of functional antibody activity, such as neutralisation capacity, which provides important information on the biological relevance of antibody responses. Moreover, the PExA samples do not include immune cells, which are a critical component of the immune response. However, cellular immune responses may be partially assessed by measuring soluble biomarkers associated with T-cell activity, including cytokines, chemokines, and cytotoxic effector molecules.^46^ By integrating biomarker profiling with antibody measurements, future studies could expand the utility of PExA to provide a more comprehensive understanding of mucosal immunity, encompassing both humoral and cellular responses, without the need for invasive sampling. Additionally, the sample size was too small to allow for stratified analyses exploring the relative contribution of infection- and vaccine-induced antibody responses. The predominance of female sex in the study cohort precluded meaningful sex-stratified analyses and may limit the generalisability of the findings to broader populations.

Several non-invasive airway sampling methods exist, such as induced sputum and exhaled breath condensate (EBC), yet both have notable limitations. Induced sputum is susceptible to upper-airway contamination and mainly reflects larger and more proximal airways, which represent central rather than distal airways.^47^ Direct comparisons between induced sputum and PExA samples have, to our knowledge, not yet been reported. As the two methods sample distinct anatomical compartments of the respiratory tract, differences in molecular composition are to be expected. EBC is highly sensitive to collection conditions, prone to contamination, and inefficient for capturing low-abundance non-volatile molecules.^48^ As with sputum, EBC also has an unknown and variable dilution factor which complicates biomarker normalisation, and it does not reliably originate from the distal airways.

In conclusion, this study provides evidence that antigen-specific antibodies can be quantified non-invasively in the lower airways using particles in exhaled air. This approach may overcome a long-standing obstacle to assessing lower-airway immunity, enabling repeated sampling and longitudinal characterisation of local antibody kinetics. Further studies are needed to establish its broader applicability and potential utility in clinical or interventional research settings, including the evaluation of next-generation mucosal vaccines.

## Contributors

Katherina Aguilera: Writing: original draft, Writing: review & editing, Study design, Methodology, Formal analysis, Investigation, Data curation, Visualisation, Resources.

Andreas Hober: Visualisation, Formal analysis. Writing: review & editing, Investigation. Validation.

Nina Greilert Norin: Investigation, Resources, Writing: review & editing.

Mina Mahdi: Investigation, Writing: review & editing.

Vera C Stäheli: Investigation, Writing: review & editing.

Iryna Kolosenko: Methodology, Writing: review & editing.

Pia Lindberg: Methodology, Writing: review & editing.

Emma Larsson: Investigation, Resources, Writing: review & editing.

Ali Rihani: Methodology, Investigation, Writing: review & editing.

Jessica J Alm: Methodology, Investigation, Writing: review & editing.

Åsa M. Wheelock: Methodology, Writing: review & editing.

Jonas Klingström: Investigation, Supervision, Writing: review & editing.

Tamás Pongrácz: Visualisation, Formal analysis. Writing: review & editing, Validation.

Mikael Åberg: Writing: review & editing, Resources, Methodology, Data curation, Supervision.

Charlotte Thålin: Writing: review & editing, Supervision, Resources, Project administration, Funding acquisition, Conceptualisation, Data verification. All authors critically revised the manuscript and approved the final version submitted for publication. Charlotte Thålin (corresponding author) and Katherina Aguilera accessed and verified the underlying data reported in this manuscript.

## Data sharing statement

Pseudonymised individual participant data underlying the findings reported in this article, including raw and analysed data and the corresponding data dictionary, will be made available to qualified researchers upon reasonable request to the corresponding author (C.T.; charlotte.thalin@ki.se), beginning upon publication and for up to 10 years thereafter. Requests will be reviewed by the corresponding author (C.T.) and will be made available for scientifically sound research purposes, subject to applicable ethical approvals and a data-use or material transfer agreement, as appropriate. Individual participant-level data cannot be deposited in a public repository because they contain sensitive personal data and are subject to applicable data-protection and confidentiality requirements. The study protocol, analysis plan, and analytic files will be available upon reasonable request where permitted. No custom code or scripts were used. Enquiries regarding data availability should be directed to the corresponding author (C.T.; charlotte.thalin@ki.se).

## Declaration of interests

Åsa Wheelock is a Scientific Advisor to PExA AB. The remaining authors declare no competing interests. PExA AB kindly provided a PExA instrument without charge during the study period.

PExA AB has filed a patent relating to methods of quantifying antigen-specific antibodies in PExA samples which lists Charlotte Thålin and Mikael Åberg as inventors.
