# Supplementary material for: Non-invasive quantification of lower airway antigen-specific antibodies using exhaled breath particles – an observational proof-of-concept study
Source: eBioMedicine. 2026 Nov 2;133:106538. doi: 10.1016/j.ebiom.2026.106538 (PMC13755983; doi:10.1016/j.ebiom.2026.106538)
Supplement: Supplementary file 1 [file mmc1.docx]

**Supplementary Appendix**

## **Non-invasive quantification of lower airway antigen-specific antibodies using exhaled breath particles – an observational proof-of-concept study.**

Katherina Aguilera, MSc^1^, Andreas Hober, PhD^1^, Nina Greilert Norin, RN^1^, Mina Mahdi, MD^1^, Vera C Stäheli, BSc^1^, Iryna Kolosenko, PhD^2,3^, Pia Lindberg, RN^2,3^, Emma Larsson, BSc^1^, Ali Rihani, BSc^4^, Jessica J Alm, PhD^4,5^, Åsa M. Wheelock, PhD^2,3^, Jonas Klingström, PhD^6^ , Tamás Pongrácz, PhD^1^, Mikael Åberg, PhD^7^*, Charlotte Thålin, MD, PhD^1^*#

**Table of contents**

Methods	1

RNA Extraction and RT-qPCR Analysis	1

Supplementary Figure S1	3

Supplementary Table S1	4

Supplementary Table S2	5

**RNA Extraction and RT-qPCR Analysis**

Three-point (throat, nasal, saliva) swab samples were self-collected at symptom onset and analyzed by real-time PCR for SARS-CoV-2, influenza, and RSV viruses at the National Pandemic Center (NPC), Karolinska Institutet, using validated diagnostic protocols. RNA extraction and RT-qPCR analyses were performed using automated magnetic bead–based extraction and in-house one-step multiplex assays targeting influenza A (pan-INFA and H1N1), RSV (pan A and B) and human RNase P, as well as the Sansure IVD kit for SARS-CoV-2 detection.

For influenza and RSV virus detection, RNA was extracted from 180 µL of each sample using the MGI Viral DNA/RNA Extraction Kit (Cat: 1000020261, MGI Tech, China) on an automated magnetic bead–based platform. Extracted RNA was analyzed with in-house one-step RT-qPCR assays targeting pan-Influenza A (INFA), Influenza A H1N1, RSVA/B and human RNase P (internal control). Reactions were performed in duplicate using the Reliance One-Step Multiplex Supermix (Cat: 12010221, Bio-Rad, USA) with ROX (INFA), FAM (H1N1), HEX (RSVA/B) and Cy5 (RNase P) reporter dyes on a Bio-Rad CFX384 system. Cycling conditions were 50 °C for 30 min, 95 °C for 1 min, followed by 44 cycles of 95 °C for 15 s and 60 °C for 30 s with fluorescence acquisition at 60 °C.

Primers and probes for the in-house assays were synthesized by Integrated DNA Technologies (IDT, Coralville, IA, USA). Exact sequences are not disclosed due to ongoing IP protection efforts; however, contextual information is provided in accordance with MIQE (Minimum Information for Publication of Quantitative Real-Time PCR Experiments) guidelines (PMID: 21421813). The **H1N1 assay** targets the hemagglutinin (HA) segment 4 (KR611222.1), with an anchor nucleotide at position 101 and a context sequence length of 239 bp. The **pan-Influenza A assay** targets the matrix protein (segment 7; PP576969.1), with an anchor nucleotide at position 40 and a context sequence length of 178 bp. The RSV assay targets non structural protein 1 with an anchor nucleotide at position 80 and a context sequence length of 198 bp, the **human RNase P assay** targets NM_001104546.2, with an anchor nucleotide at position 39 and a context sequence length of 88 bp. Melting temperatures (Tm) and GC content for the primers and probes were designed to optimize amplification efficiency and specificity. The anchor nucleotide is defined as a nucleotide contained anywhere within the probe sequence. The context sequence length represents the amplicon plus surrounding bases as recommended by MIQE guidelines for primer/probe disclosure. Positive control of H1N1 consisted of genomic RNA of Influenza A H1N1 virus (ATCC VR-1520; American Type Culture Collection, Manassas, VA, USA).

In parallel, SARS-CoV-2 RT-qPCR was performed in duplicate using the Sansure IVD one-step RT-qPCR kit (Cat: S3102E, Sansure Biotech, China), targeting ORF1ab and N genes with human RNase P as an internal control. Reactions were run in 96-well plates on a CFX96 real-time PCR system (RRID:SCR_018064, Bio-Rad Laboratories, Hercules, CA, USA) as previously described (Hober A et al 2021 PMID: 34747696, Marking et al 2023 PMID 36949041). Each plate included a no-template control, a positive control from the kit, and two extraction blanks. Data were analyzed using CFX Maestro software (Bio-Rad, USA).

**Figure S1.** Flow chart of participant recruitment. Participants were selected for this substudy, from the ongoing COMMUNITY HCW cohort, based on a home-based PCR screening program during the spring of 2025. A total of 18 participants tested positive for SARS-CoV-2, influenza virus and/or RSV and were included in this study (two tested positive for SARS-CoV-2, eight tested positive for influenza A (H1N1) virus infection, two tested positive for influenza B virus infection, four tested positive for RSV, and two tested positive for both SARS-CoV-2 and influenza A virus or RSV with intervals of 3 and 1 month, respectively). In addition to infected participants, 10 participants without known infection in the previous 6 months were included from the COMMUNITY cohort, rendering a total of 28 study participants.

* Two participants with influenza B infection and one participant with ongoing SARS-CoV-2 infection (positive RT-qPCR at baseline) were excluded from the infection-induced antibody analysis presented in Figure 2A

**Table S1.** **Mechanistic and Biological Support for PExA Particle Origin**

| Evidence Category | Statement / Claim | Key Supporting References | Observations consistent with origin from distal airways |
| --- | --- | --- | --- |
| Biological composition | PEx particles originate from distal airway lining fluid (RTLF). | Almstrand et al., 2010; Bake et al., 2019; Bredberg et al., 2012; Behndig et al., 2019 | Surfactant phospholipids (e.g., DPPC) and lung proteins detected in PEx particles are characteristic of distal airway lining fluid. |
|  | Particle composition excludes saliva or upper airway contamination. | Bredberg et al., 2012; Bake et al., 2019 | Absence of salivary markers such as α‑amylase and airway mucins (MUC5AC/MUC5B) indicates particles are not derived from oral or upper airway sources. |
| Physiological experiments | Airway closure and reopening drives particle generation. | Almstrand et al., 2010; Holmgren et al., 2013; Larsson et al., 2017; Groma et al., 2025; Morawska et al., 2009, Schumm et al., 2023; Morawska et al.,2022 | Particle counts increase after airway closure maneuvers and during reopening of small airways, indicating reopening events trigger aerosol generation. |
| Aerosol modeling | Fluid film rupture in bronchioles is a plausible droplet generation mechanism. | Huang et al., 2020; Gumulya et al., 2023; Lv et al., 2025; Erken et al., 2022; Morawska et al.,2022 | Fluid mechanics experiments and simulations show that reopening liquid-lined airways can rupture mucus–surfactant films and produce micron-sized droplets. |
| Airway surface film properties | Particle yield depends on mucus and surfactant film properties. | Halpern, 2010; Halpern/Fujioka et al., 2008 | Film stability and viscoelastic properties influence rupture probability and aerosol yield, explaining variability between individuals. |
| Method validation | The PExA method selectively samples distal airway particles. | Roe et al., 2024 | Methodological reviews conclude that the PExA platform captures aerosol originating from distal airways and enables non-invasive sampling of airway lining fluid. |
|  | PExA sampling is feasible across a wide range of populations and disease severities. | Soares et al., 2018; Hussain-Alkhateeb et al., 2021 | PExA method is feasible and reliable across a spectrum of disease severity with high completion rates of sampling procedure and sufficient particle materials for analysis. |

**Table S2. Demographics of the study population**

|  | SARS-CoV-2^*^ | Influenza A^*^ | RSV^*^ | Non-infected^**^ | All** |
| --- | --- | --- | --- | --- | --- |
| **Number of individuals** | 3^##^ | 9^#^ | 5^#^ | 10 | 28 |
| **Mean age (SD)** | 51 (2) | 50 (8) | 53 (10) | 52 (12) | 50 (11) |
| **Female sex** | 3 (100%) | 8 (89%) | 5 (100%) | 9 (90%) | 26 (93%) |
| **Male sex** | 0 (0%) | 1 (11%) | 0 (0%) | 1 (10%) | 2 (7%) |
| **Mean days from last SARS-CoV-2 vaccine to baseline sample (SD)** | 1350 (58) | 1320 (73) | 1380 (55) | 1270 (111)*** | 1320 (84) |
| **Mean days from last influenza vaccine to baseline sample (SD)** | 365 | 318 (140) | 375 (9) | 232 (202) | 305 (148) |
|  | Missing: 2 | Missing: 3 | Missing: 1 | Missing: 5 | Missing: 13 |

Infection groups are not mutually exclusive because participants with sequential infections contribute to more than one infection-specific group.

*Refers to dataset in Figure 2A-B, and contributes to datasets in Figures 3-5.

**Refers to dataset in Figure 2B, and contributes to datasets in Figures 3-5

*** Contributes to datasets in Figures 3-5

^#^ One of these participants had a subsequent SARS-CoV-2 infection.

^##^ Two of these participants had a prior infection (RSV or influenza).
